# Combinatorial epigenetic therapy in diffuse large B cell lymphoma pre-clinical models and patients

**DOI:** 10.1186/s13148-016-0245-y

**Published:** 2016-07-22

**Authors:** Benet Pera, Tiffany Tang, Rossella Marullo, Shao-Ning Yang, Haelee Ahn, Jayeshkumar Patel, Rebecca Elstrom, Jia Ruan, Richard Furman, John Leonard, Leandro Cerchietti, Peter Martin

**Affiliations:** Division of Hematology and Medical Oncology, Weill Cornell Medical College and New York Presbyterian Hospital, New York, NY 10065 USA; National Cancer Center Singapore, Singapore, 169610 Singapore; Present address: Genentech, Inc., 1 DNA Way, 444B, San Francisco, CA 94080 USA; Hematology and Oncology Division, Weill Cornell Medical College, Cornell University, 1305 York Ave, New York, NY 10065 USA

**Keywords:** Histone deacetylase inhibitors, Demethylating agents, Lymphoma, Chemosensitization, Phase Ib

## Abstract

**Background:**

Refractory and/or relapsed diffuse large B cell lymphoma (RR-DLBCL) patients are incurable with conventional chemotherapy due to the aggressiveness and the chemorefractory state of these tumors. DNA hypermethylation and histone deacetylation are two major epigenetic modifications by which aggressive DLBCL maintain their oncogenic state. We have previously reported that DNA methyltransferase inhibitors (DNMTI) affect RR-DLBCL growth and improve chemosensitivity. Here, we hypothesized that the combination of DNMTI with histone deacetylase inhibitor (HDI) would be an active and feasible therapeutic strategy in RR-DLBCL. Thus, we evaluated the anti-lymphoma activity of the HDI vorinostat (VST) in combination with the DNMTI azacitidine (AZA) or decitabine (DAC) in pre-clinical models of RR-DLBCL, and we determined the feasibility of the combination by conducting a phase Ib trial in RR-DLBCL patients.

**Results:**

Concurrent combination of DNMTI and HDI resulted in synergistic anti-lymphoma effect toward RR-DLBCL cells in vitro and in vivo, with no significant toxicity increase. In a phase Ib trial, a total of 18 patients with a median of three prior therapies were treated with four different dose levels of AZA and VST. The most common toxicities were hematological, followed by gastrointestinal and metabolic. The clinical benefit was low as only one subject had a partial response and three subjects had stable disease. Interestingly, two of the seven patients that received additional chemotherapy post-study achieved a complete response and three others had a significant clinical benefit. These observations suggested that the combination might have a delayed chemosensitization effect that we were able to confirm by using in vitro and in vivo models. These studies also demonstrated that the addition of VST does not improve the chemosensitizing effect of DAC alone.

**Conclusions:**

Our data supports the strategy of epigenetic priming by employing DNMTI in RR-DLBCL patients in order to overcome resistance and improve their outcomes.

**Electronic supplementary material:**

The online version of this article (doi:10.1186/s13148-016-0245-y) contains supplementary material, which is available to authorized users.

## Background

Diffuse large B cell lymphoma (DLBCL) is the most common form of non-Hodgkin lymphoma in adults. DLBCL is an aggressive but potentially curable disease as roughly 60 % of patients obtain a complete and sustained response with the standard chemo-immunotherapy treatment of the anti-CD20 monoclonal antibody rituximab and four chemotherapeutic agents (cyclophosphamide, doxorubicin, vincristine, and prednisolone) (RCHOP) [1, 2]. Unfortunately, the prognosis of patients with primary chemotherapy refractory disease or those that relapse after initial response remains poor. Response rates to second-line chemotherapy regimens are roughly 50 % with long-term survival rates of only 20 % despite aggressive approaches such as autologous stem cell transplantation [3]. Patients that do not respond to second-line therapy have an average survival of only 4 months [4]. Thus, novel approaches are needed for patients with relapsed or refractory DLBCL (RR-DLBCL). Epigenetic modifications such as DNA methylation and histone acetylation orchestrate the regulatory state of chromatin. Many cancers display global changes in histone modifications and aberrant methylation patterns that allow them to sustain their oncogenic state. Malignant cells repress the expression of genes involved in tumor suppressive pathways mostly through DNA hypermethylation and histone deacetylation. DNA methyltransferases (DNMT) and histone deacetylases (HDAC) are the key enzymes leading this process [5]. Since epigenetic silencing is a reversible process, pharmacological inhibition of DNMTs and HDACs has rapidly became an attractive therapeutic strategy as it allows the restoration of those non-functional pathways in tumor cells inducing phenotypic changes that may lead to cell death. Interestingly, DLBCL patients with more aggressive disease exhibit a greater degree of epigenetic aberrations [5, 6]. Moreover, our group has previously demonstrated that administration of DNMT inhibitors (DNMTI) induces reprogramming of refractory DLBCL cells resulting in recovery of sensitivity toward DNA damaging agents [7]. Overall, these data suggest that aggressive RR-DLBCL may rely on epigenetic silencing of multiple genes to survive as well as tolerate genotoxic stress. Epigenetic drugs such as HDAC inhibitors (HDI) and DNMTI have shown to be well tolerated but with limited activity in DLBCL patients [8, 9]. The modest activity of these drugs as single agents suggests that pathways critical for survival may be simultaneously regulated by different epigenetic modifications in DLBCL. This hypothesis is supported also by the observation that hypermethylated CpG islands attract methyl-CpG-binding domain proteins (MBDPs) and recruit HDACs, leading into an enhanced transcriptional inhibition [10, 11]. Thus, the combination of HDI and DNMTI may achieve a greater efficacy in disrupting the transcription repressor complex composed by MBDPs and HDACs. We therefore speculated that combinatorial targeting of HDAC and DNMT could (i) exert synergistic cytotoxic effect on RR-DLBCL; (ii) be a safe and active therapeutic strategy in patients with aggressive RR-DLBCL; (iii) achieve a greater degree of chemosensitization compared to DNMTI alone in RR-DLBCL. Following this rationale, first we determined if the combination of the DNMTI and HDI would yield a better anti-lymphoma effect in pre-clinical models of RR-DLBCL. We then evaluated the feasibility of this combinatorial treatment by conducting a phase Ib study of the DNMTI 5-azacitidine (azacitidine, AZA) with the HDI suberoylanilide hydroxamic acid (vorinostat, VST) in patients with RR-DLBCL. Finally, by using in vitro and in vivo pre-clinical models, we explored whether the simultaneous inhibition of DNMT and HDAC may enable RR-DLBCL to regain chemosensitivity.

## Methods

### Drugs and reagents

5-aza-2′-deoxycytidine (decitabine, DAC) was from Dacogen, 5-azacitidine (azacitidine, AZA) was from Aton Pharma, and suberoylanilide hydroxamic acid (vorinostat, VST) was from LC Laboratories. All three drugs were dissolved in distilled water and used within an hour of preparation.

### Cell lines

Human DLBCL cell lines OCI-Ly1, OCI-Ly4, and OCI-Ly7 were grown in medium containing 90 % Iscove’s modified Eagle medium and 10 % fetal calf serum (FCS; 20 % FCS for OCI-Ly7) supplemented with 1 % penicillin G and streptomycin. DLBCL cell lines DB, Farage, HT, Karpas422, Karpas231, NU-DUL-1, OCI-Ly3, OCI-Ly8, OCI-Ly18, OCI-Ly19, OZ, RL, RC-K8, RI-1, SU-DHL4, SU-DHL5, SU-DHL6, SU-DHL7, SU-DHL8, SU-DHL10, SC1, Toledo, VAL1, and WSU-NHL were grown in medium containing 90 % RPMI-1640 and 10 % FCS supplemented with 1 % penicillin G and streptomycin, 1 % l-glutamine, and 1 % HEPES. Cells were maintained in a 37 °C, 5 % CO_2_, fully humidified incubator. All cell lines were purchased from the American Type Culture Collection, Leibniz Institute DMSZ-German Collection of Microorganisms and Cell Cultures, or the Ontario Cancer Institute. Monthly testing for *Mycoplasma sp*. and other contaminants and quarterly cell identification by single-nucleotide polymorphism were conducted.

### Growth inhibition determination

All cell lines were grown at concentrations that assured to maintain non-treated cells in exponential growth over the drug exposure time. Cell viability was determined by a fluorescence assay using resazurin (7-Hydroxy-3H-phenoxazin-3-one 10-oxide) (CellTiter-Blue, Promega). Fluorescence was measured in a Synergy4 microplate reader (BioTek) employing 560 and 590 nm of excitation and emission wavelengths, respectively. The number of viable cells was calculated by using the linear least-squares regression of the standard curve. The fluorescence emission was determined for three replicates per treatment condition and normalized to their respective controls (vehicle-treated cells). Dose-effect curves were plotted, and GI50 values were determined using GraphPad Prism version 6.0b software. Data were presented as the mean GI50 with a 95 % confidence interval for duplicate experiments.

### Drug combination analysis

Interaction between vorinostat and decitabine was evaluated employing the combination index (CI) equation of Chou and Talalay [12]: CI = (D_1_/Dx_1_) + (D_2_/Dx_2_). A CI value equal to one indicates additivity, values less than one indicate synergy, and values greater than one indicate antagonism. Doses D_1_ and D_2_ correspond to those used in combination, and the doses Dx_1_ and Dx_2_ correspond to the amounts of each drug given alone that would produce the same response as obtained with the combination. CI values were calculated employing CompuSyn software, and isobolograms were plotted using GraphPad Prism version 6.0b software.

### Mice studies

All mouse procedures were approved by The Research Animal Resource Center of the Weill Cornell Medical College of Medicine. Adult (6- to 8-week-old, male, weighting average of 20 g) severe combined immunodeficiency (SCID) mice were purchased from the US National Cancer Institute (NCI) and subcutaneously injected in the left flank with 10 × 10^6^ low-passage human RR-DLBCL OCI-Ly7 cells. Tumor size was monitored every other day employing electronic digital calipers. Tumor volume was calculated using the equation: tumor volume = (smallest diameter^2^ × largest diameter)/2. Treatment schedules are described in the “Results” section. Decitabine and vorinostat were dissolved in 100 % sterile water and 1:1 sterile water in DMSO, respectively. Both drugs were administered intraperitoneally. The mice were weighed every other day. At the end of the experiment, the mice were euthanized by CO_2_ inhalation.

### Clinical trial design

An open-label phase Ib study in patients with relapsed or refractory DLBCL was approved by the Institutional Review Board of Weill Cornell Medical College (ClinicalTrials.gov identification number NCT01120834). The primary objective was to determine the safety and tolerability of the combination of AZA (Celgene) with VST (Merck) and to determine an appropriate dose for further evaluation. Secondary objectives were to determine the efficacy of this combination by measuring the overall response rates (ORR) and overall survival (OS). We performed a retrospective review of seven subjects who proceeded to have treatment post-study and evaluated their ORR and subsequent OS following the study regimen.

#### Patients

Eligible patients were >18 years of age and had histologically confirmed RR-DLBCL. Patients had to have measurable disease and had to be deemed ineligible for autologous stem cell transplantation. There was no limit on the number of prior therapies and previous autologous stem cell transplantation was allowed. Patients also had to have an Eastern Cooperative Oncology Group (ECOG) performance score ≤2, absolute neutrophil count (ANC) ≥1000/μL, platelets ≥75/μL, and adequate renal and hepatic function. Exclusion criteria included a prior allogeneic transplant, previous HDAC inhibitors for anti-lymphoma therapy, active central nervous system (CNS) lymphoma, active second malignancy, uncontrolled illness, and a QTc interval of >0.470.

#### Treatment

AZA was administered via subcutaneous injection, and VST was taken orally at four different dose levels (DL): AZA 55 mg/m^2^ days 1–5 and VST 300 mg BID days 1–7 (DL1), AZA 75 mg/m^2^ days 1–5 and VST 200 mg BID days 1–7 (DL2), AZA 55 mg/m^2^ days 1–5 and VST 300 mg BID days 1–14 (DL3), and AZA 75 mg/m^2^ days 1–5 and VST 200 mg BID days 1–14 (DL4). Each cycle was 28 days, and patients were treated for up to six cycles. If at any time two patients in a given cohort experienced dose-limiting toxicities (DLT), enrollment to that level was discontinued. Up to eight patients were enrolled to each dose level, and accrual to the next dose level was dependent on the toxicity and efficacy of the previous dose levels. Each of these doses had been explored in phase I trials of myelodysplastic syndrome (MDS), and all had been found to be tolerable.

#### Assessments

Tumor assessments were made with ^18^FDG-PET/CT (fluorodeoxyglucose-18 positron emission tomography/computed tomography) scans prior to the third cycle and after the sixth cycle of treatment and CT scans were planned at 3-monthly intervals during post-treatment follow-up evaluations. Treatment responses were defined according to the International Workshop Criteria (IWC) [13] and the revised International Workshop Criteria with integration of ^18^FDG-PET (IWC + PET) [14] and classified as either complete response (CR), CR unconfirmed (CRu), partial response (PR), stable disease (SD), or progressive disease (PD). Safety was assessed by adverse events (AE) graded according to the National Cancer Institute Common Terminology Criteria for Adverse Events version 4.0 with DLT defined as any of the following treatment-related AE during the first cycle of treatment: grade 3–4 non-hematologic toxicity excluding alopecia, nausea, vomiting, or fatigue responding to maximal treatment; any grade 4 hematological toxicity including grade 4 neutropenia lasting longer than 7 days in the setting of G-CSF use; failure of ANC to recover to >1000 cells/μL or platelets to recover to >50,000/μL within 14 days; and grade 4 thrombocytopenia of any duration. Febrile neutropenia <grade 4 was not considered a DLT.

#### Statistical analysis

Safety data and response rates were summarized using descriptive statistics. OS was calculated from date of study entry to the time of death from any cause or the date of last follow-up. Data on patients alive at last follow-up were censored during the survival analysis. A post hoc review was performed on patients who received other treatment after study discontinuation. Time to progression (TTP post-study) was calculated from the date off study to the date of progression and OS post-study was calculated from the date off study to the date of death or last follow-up.

## Results

### Vorinostat and decitabine combination shows synergistic anti-lymphoma effect on DLBCLs

**Fig. 1:**
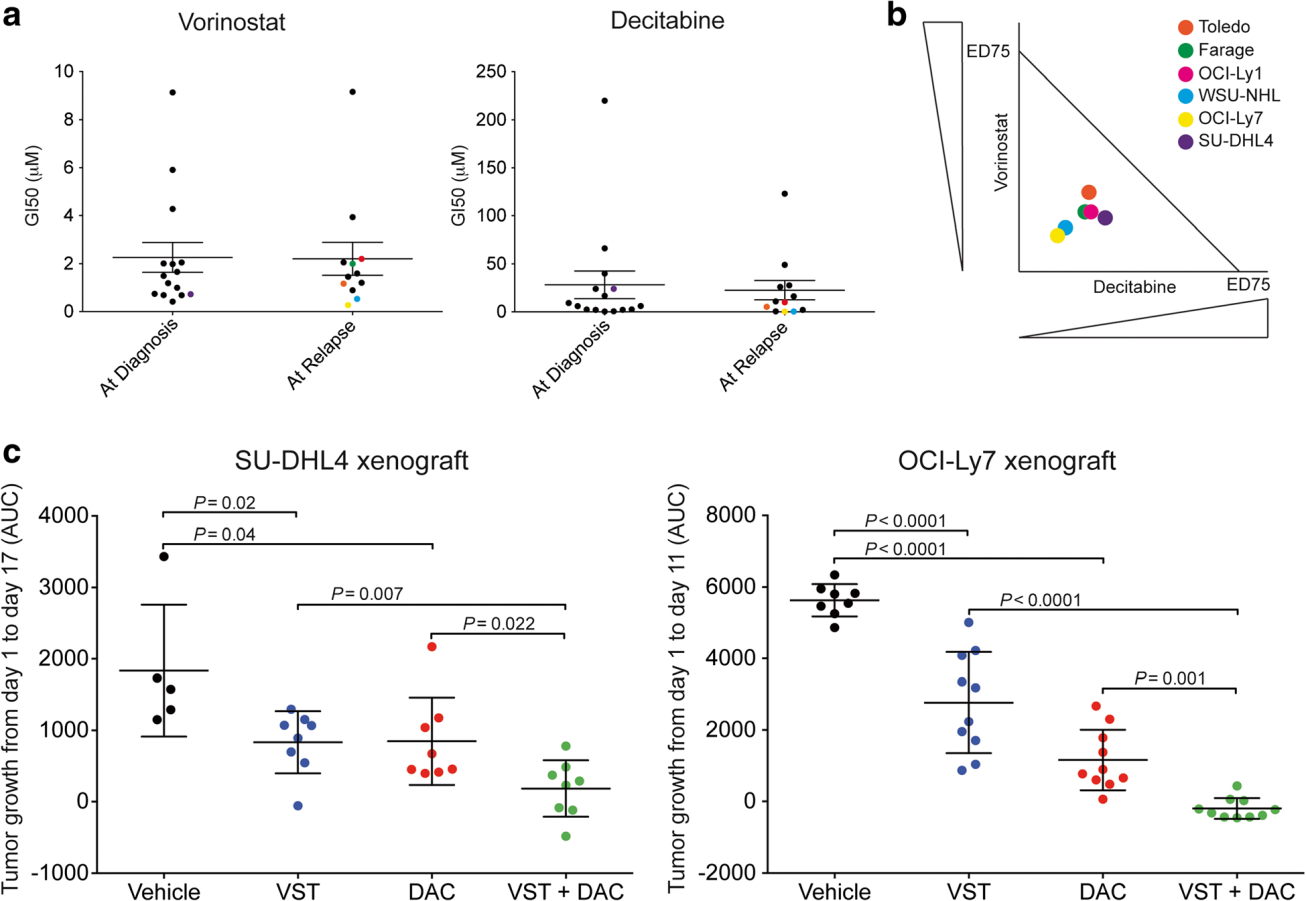
Effect of the combination vorinostat with decitabine on DLBCL cells. **a** Scatter plots of the GI50 values of vorinostat (VST, *left*) and decitabine (DAC, *right*) in a panel of 27 DLBCL cell lines obtained at initial diagnosis or at relapse. **b** Isobologram for Toledo, Farage, OCI-Ly1, WSU-NHL, OCI-Ly7, and SU-DHL4, tested for the combination of DAC and VST. *Circles* represent each cell line with their relative position to the additive line indicating the result from the combination. Cells below the line indicate a synergistic effect. **c** DLBCL tumor growth curves (shown as area under the curve) in SU-DHL4 (*left*) and OCI-Ly7-xenografted (*right*) mice treated with vehicle, VST, DAC, or VST + DAC

To perform combinatorial studies of epigenetic drugs in relapsed DLBCL cell lines, we first determined the sensitivity to vorinostat and decitabine in a panel of 27 DLBCL cell lines, 15 of them established at diagnosis (P-DLBCL) and 12 of them established from relapsed patients after at least one line of treatment (R-DLBCL). We have previously showed that R-DLBCL cell lines remained resistant to at least one of the chemotherapy agents included in the CHOP regimen (i.e., doxorubicin, vincristine, mechlorethamine for cyclophosphamide, and dexamethasone for prednisone) [7]. We exposed this panel to vorinostat or decitabine for 48 h and determined their proliferation activity by a resazurin reduction assay. The administration of vorinostat or decitabine reduced the proliferation of all tested cell lines regardless their origin (Fig. 1a), suggesting that, despite their origin, R-DLBCL are similarly sensitive to these epigenetic drugs. We then selected five R-DLBCL (OCI-Ly1, OCI-Ly7, WSU-NHL, Farage and Toledo) and P-DLBCL (SU-DHL4) cell lines to determine the anti-proliferative effect of the combination of vorinostat with decitabine by calculating the CI [12]. Similar to individual drugs, the combination of vorinostat and decitabine was synergistic in all the R-DLBCL and P-DLBCL cell lines tested (Fig. 1b).

To determine whether the combination of vorinostat and decitabine is effective in R-DLBCL in vivo, we administer human-equivalent doses of vehicle, vorinostat 20 mg/kg/day, decitabine 15 mg/m^2^/day, or their combination to mice bearing R-DLBCL OCI-Ly7 cell line xenografts. We used P-DLBCL SU-DHL4 xenografts as controls. When xenografts reached 75 to 100 mm^3^, the mice were randomized in four groups and treated with vehicle, decitabine, vorinostat, or the combination of vorinostat + decitabine, daily for 11 and 17 days in OCI-Ly7 and SU-DHL4 xenografts, respectively (Fig. 1c). The mice were followed until untreated tumors reached 1500 mm^3^. Compared to the vehicle-treated mice, decitabine or vorinostat alone significantly suppressed the growth of R-DLBCL OCI-Ly7 and P-DLBCL SU-DHL4 xenografts. In both models, the combination of vorinostat + decitabine resulted in a significantly higher lymphoma control than either drug alone. There was no evidence of toxicity to normal tissues based on body weight and macroscopic examination of tissues at necropsy. Overall, these data suggest that the combination of vorinostat and decitabine may be effective toward R-DLBCL with no evidence of gross toxicity.

### Phase Ib study of azacitidine and vorinostat in patients with relapsed or refractory DLBCL

The promising results observed in pre-clinical model prompted us to perform a phase Ib study (Table 1) to evaluate the combination of the DNA methyltransferase inhibitor azacitidine and the histone deacetylase inhibitor vorinostat. Subjects with RR-DLBCL ineligible (due to chemorefractory state or comorbidities) or relapsed after autologous stem cell transplant were treated with azacitidine and vorinostat at four different dose levels (DL). A total of 18 patients were enrolled (Table 2). The median age was 66 years old. The subjects had received a median of 3 prior lines of treatment, including three that had undergone autologous stem cell transplantation. Thirteen patients were refractory to their previous treatment. Eight patients were treated at dose level 1 (DL1), five at DL2, four at DL3, and one at DL4. DL3 and DL4 were closed early after two patients at DL3 required dose reductions. A median of two cycles (range 1–6) of treatment was administered. One patient completed 6 cycles of treatment, and 17 patients stopped treatment due to PD. The trial was terminated prematurely because of low clinical activity and poor tolerability at the doses/schedules tested in this study.

**Table 1:** Overview of the phase Ib study on the combination of vorinostat and azacitidine in RR-DLBCL patients

Open-label phase Ib (NCT01120834)			
Key enrollment criteria	Dose level 1	Key endpoints	Post-study
R/R-DLBCL	AZA 55 mg/m^2^ s.q. D 1 to 5	Primary: MTD or MAD	ORR and subsequent OS
	VST 300 mg/m^2^ BID D 1 to 7		
Relapsed after or ineligible for ASCT	Dose level 2		
ANC > or = 1000/μL	AZA 75 mg/m^2^ s.q. D 1 to 5	Secondary: Efficacy	
	VST 200 mg/m^2^ BID D 1 to 7		
Platelets > or = 75/μL	Dose level 3		
Adequate renal and hepatic functions	AZA 55 mg/m^2^ s.q. D 1 to 5		
	VST 300 mg/m^2^ BID D 1 to 14		
No prior treatment with HDACIs	Dose level 4		
QTc no more than 0.470	AZA 75 mg/m^2^ s.q. D 1 to 5		
	VST 200 mg/m^2^ BID D 1 to 14

**Table 2:** Baseline clinical characteristics of the subjects enrolled into the study

	All patients (*n* = 18)	
Characteristic	Number	%
Gender		
Male	11	61
Female	7	39
Age in years		
Median (range)	66.5 (27–83)	
ECOG		
0	0	0
1	10	55.6
2	8	44.4
Histological diagnosis		
DLBCL	15	83.3
Transformed FL	2	11.1
CLL with Richter’s transformation	1	5.5
Prior treatments regimens		
Median (range)	3 (1–4)	
Rituximab-containing	18	100
Autologous stem cell transplantation	3	16.7
Response to prior line of therapy		
CR	4	22.2
PR	1	5.6
Refractory	13	72.2

The following grade 1–2 hematological and non-hematologic toxicities, independent of relation to study drugs, were experienced by at least two subjects: anemia (*n* = 14), thrombocytopenia (*n* = 6), leucopenia (*n* = 5), neutropenia (*n* = 2), nausea (*n* = 12), hypoglycemia (*n* = 7), renal impairment (*n* = 7), diarrhea (*n* = 6), vomiting (*n* = 6), raised ALP (*n* = 5), hyperglycemia (*n* = 5), fatigue (*n* = 4), fever (*n* = 3), and hyperbilirubinemia (*n* = 3). One patient experienced a grade 3 thromboembolism at DL1, one experienced grade 3 diarrhea at DL2, and one experienced grade 4 increase in ALP at DL4. Grade 3–4 hematologic toxicity included thrombocytopenia (*n* = 8), anemia (*n* = 3), and neutropenia (*n* = 2) (Additional file 1: Table S1). Only one DLT occurred at DL1 as a result of grade 4 thrombocytopenia.

At DL1, two subjects had SD, five had PD, and response status was not evaluable in two patients. At DL2, one subject had a PR, one had SD, and three had PD. At DL3, three had PD, and response status was not evaluable in one. The only subject treated at DL4 had PD (Additional file 2: Table S2). Hence, the ORR was 6.7 % (1/15), and 86.7 % (13/15) had PD in evaluable subjects. The 3-month OS for the entire cohort was 77 % (95 % CI 34–94 %).

### Clinical follow-up suggests a potential chemosensitization effect of the combination of DNMTI and HDI

Seven subjects enrolled in the phase Ib study received further treatment. Prior to enrolling in the trial, two patients had relapsed disease and five had disease that was refractory to last pre-study therapy. These subjects received a median of two cycles of azacitidine and vorinostat. Subsequent therapies included veltuzumab radioimmunotherapy (*n* = 1), oral PEP-C (procarbazine, etoposide, prednisone, cyclophosphamide; *n* = 2), brentuximab vedotin-PEP-C (*n* = 1), and RDICE (rituximab, dexamethasone, ifosfamide, cisplatin, etoposide) followed by allogeneic stem cell transplantation (*n* = 1) and bendamustine (*n* = 2).

Although one of the two patients that received bendamustine achieved significant clinical benefit, the short follow-up of both patients limited any conclusion based on these two cases. For the other five patients that received post-trial treatments, two patients had a CR and two others had a significant clinical benefit (Table 3). At the time of data collection, the OS post-study in these five patients ranged from 79 to 825 days. Despite these promising responses, all four deaths were due to progressive lymphoma. Taking into account the fact that the majority of these patients had disease that was refractory to the last therapy, these data suggest that combination of vorinostat and azacitidine, although providing only marginal disease control during administration, was associated with a delayed chemosensitization effect.

**Table 3:** Follow-up details of subjects who had further treatment after the phase Ib study

Patient	Prior therapies	Response to prior line	Duration of response (months)	No. of AZA-vorinostat cycles	Subsequent therapies	Time between combination to subsequent therapy (days)	Response to subsequent therapies	TTP post-study (days)	OS post-study (days)	Survival status
1	RCHOP (×6)	CR	40	3	Veltuzumab with 90Y-epratuzumab	14	Significant clinical benefit	115	682	Dead
2	RCHOP (×6), VIPER (×3), BEAM, and ASCT	PD	NA	4	Oral PEP-C	16	Significant clinical benefit	408	556	Alive
3	CHOP + RT 10 years prior. De Angelis, RDICE (×3)	CR	8	2	Oral PEP-C	18	CR	220	501	Alive
4	RCHOP (×6), RDICE, RHCVAD (Bcycle)	PD	NA	2	Brentuximab + PEP-C (×4)	2	PD	79	148	Dead
5	RCHOP (×6), ESHAP (×3), lenalidomide, ibrutinib	PD	NA	4	RDICE (×3), ASCT	11	CR	436	825	Dead
6	RCHOP (×6), RDICE (*×*2), ibrutinib	PD	NA	1	Bendamustine (×1)	1	Significant clinical benefit	NA	45	Dead
7	RCHOP (×6), RICE (*×*2), R-Nav/Gem#1	PD	NA	1	Bendamustine (×1)	4	NA	NA	35	Alive

Time to progression and overall survival calculated from the time the subject was taken off the study

*RCHOP* rituximab in combination with cyclophosphamide, doxorubicin, vincristine, and prednisolone, *VIPER* bortezomib in combination with DICE chemotherapy plus rituximab, *BEAM* combination of carmustine, etoposide, cytarabine, and melphalan, *ASCT* autologous stem cell transplant, *RT* radiotherapy, *RHCVAD* rituximab-fractionated cyclophosphamide, vincristine, adriamycin, and dexamethasone, *ESHAP* etoposide, methylprednisolone, cytarabine and cisplatin, *RCIE* rituximab, ifosfamide, carboplatin, and etoposide, *CR* complete remission, *PD* progressive disease, *NA* not available, *PEP-C* prednisone, etoposide, procarbazine, and cyclophosphamide, *TTP* time to progression, *OS* overall survival

### The chemosensitization effect of the DNMTI and HDI combination is largely due to the DNMTI

**Fig. 2:**
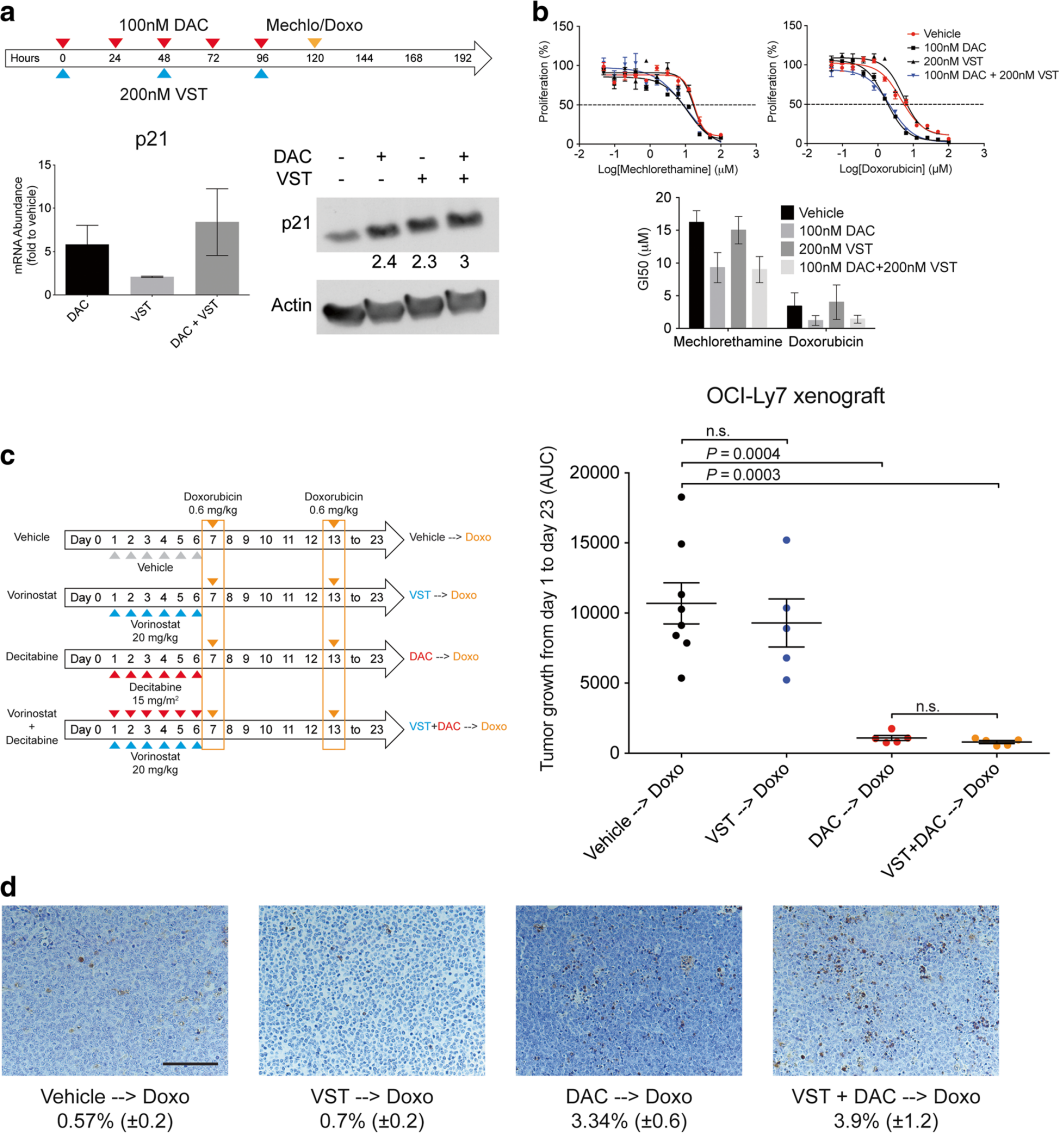
Chemosensitizing effect of the combination vorinostat with decitabine. **a** Treatment schedule performed in OCI-Ly1 cells receiving vorinostat (*VST*) and decitabine (*DAC*) followed by doxorubicin (*Doxo*) or mechlorethamine (*Mechlo*) (*upper part*), and mRNA and protein levels of p21 after 5 days of treatment (*lower part*). **b** Reduction in the growth inhibition 50 (GI50) values for mechlorethamine and doxorubicin after treatment with the VST and DAC combination. **c** Treatment schedule for mice receiving vehicle, VST, DAC, or VST + DAC, followed by doxorubicin (*left part*). On the right part, area under the curve (AUC) of tumor growth curves in OCI-Ly7-treated xenografts. **d** Representative images from OCI-Ly7 mice tumors after being treated, assayed for apoptosis by TUNEL. The percentages of stained area (mean ± the standard error) are shown at the bottom. The bar represents 100 μm

The above observation is in general agreement with our previous demonstration that DNMTI can induce reprogramming of chemorefractory DLBCL cell lines resulting in a p21-associated senescence-like phenotype and chemosensitization [7]. In the light of the clinical observation of these seven patients, and in order to determine whether the combination of DNMTI and HDI could induce a higher chemosensitizing effect than DNMTI alone, we determined the induction of p21 in OCI-Ly1 cells treated with decitabine, vorinostat or their combination. The combination of vorinostat and decitabine induced a mild increase in p21 over single agent administration (Fig. 2a). This effect was insufficient to increase the chemosensitizing effect of the combination vs. each drug alone to mechlorethamine and doxorubicin (Fig. 2b). In fact, only decitabine administration induced significant chemosensitization to both agents (Fig. 2b).

To further explore this effect, we implanted R-DLBCL OCI-Ly7 cells into SCID mice as before. When tumors developed, the mice were randomized into four groups and treated with vehicle (*n* = 8), decitabine (*n* = 5), vorinostat (*n* = 5), or the combination vorinostat + decitabine (*n* = 5), daily for 6 days. At day 7 and day 13, the mice received a dose of doxorubicin equivalent to the human dose for the treatment of lymphomas (0.6 mg/kg) (Fig. 2c). Compared to the mice that received vehicle followed by doxorubicin, the mice pre-treated with the combination of vorinostat and decitabine and the mice pre-treated with decitabine alone showed significant tumor control (*P* = 0.0003 and *P* = 0.0004, respectively). However, tumor growth in the mice that received vorinostat followed by doxorubicin were undistinguishable from those that received vehicle followed by doxorubicin (*P* = 0.556). Moreover, there was no difference in the degree of chemosensitization in mice receiving combination pre-treatment vs. the decitabine single agent pre-treatment (*P* = 0.201), suggesting that the chemosensitizing effect observed in the pre-clinical model, and potentially in patients, with the combination of vorinostat and decitabine is likely due to decitabine administration.

## Discussion

The aim of our studies was to determine whether the combination of the epigenetic drugs azacitidine and vorinostat was safe and potentially active in RR-DLBCL patients. We also aimed to evaluate in pre-clinical models whether addition of vorinostat could potentiate the chemosensitizing effect of DNMTIs previously reported by our group [7]. In agreement with previous reports [15], vorinostat and decitabine showed synergistic anti-lymphoma effect in DLBCL cell lines in vitro and in vivo. However, a similar combination evaluated in patients with RR-DLBCL was poorly tolerated and had a minimal clinical activity.

Similar studies have been performed in other hematologic malignancies [16–21]. A phase I study investigated the combination of decitabine and vorinostat in concurrent and sequential arms in 71 AML and MDS patients resulted in promising results but relatively high incidence of drug-related adverse events [16]. In the concurrent arm, which more closely mirrors the design of our study, hematologic toxicities occurred more frequently than in the sequential arm [16]. In our study, the incidence of hematological side effects was 94 % for all grades of anemia, 78 % for thrombocytopenia, 50 % for leucopenia, and 20 % for neutropenia. The higher rates of hematological side effects in our study are likely a reflection of a more heavily pre-treated cohort of patients. In another study, 40 patients with MDS were enrolled into a phase II study that tested azacitidine at 55–75 mg/m^2^ and vorinostat at 200–300 mg BID for 7–14 days at three dose levels [17]. In this study, a median of 6 cycles were administered (range 1–26), and grade 3 fatigue occurred in 32 % of patients while no grade 3 fatigue occurred in our study and this could be due to the fewer number of treatment cycles administered. In summary, we consider that combination of HDIs with DNMTIs shows limited activity in the population of patients treated in our study. Nevertheless, we consider that this combination merits further investigations in DLBCL patients with less pre-treated disease.

Despite good pre-clinical data to support the combination of azacitidine or decitabine with vorinostat, clinical responses have been dismal. One explanation for the poor clinical response observed is that the patients chosen for these clinical trials had aggressive tumors with high tumor burden that did not allow enough time for the epigenetic agents to act. In this regard, epigenetic therapy combinations with the more potent drug decitabine (instead of azacitidine) or longer administration time, for example, with oral version of these hypomethylating agents, could be worth exploring. However, our previous experience in pre-clinical models and front-line DLBCL patients treated with azacitidine suggested that tumor reduction might not be the optimal criteria to evaluate the antineoplastic effect of epigenetic drugs. While cellular reprogramming seems to be the dominant effect of these drugs on pre-clinical models [7], a validated clinical biomarker is still lacking.

Supporting the role of epigenetic drugs as chemosensitizers, we observed a potential delayed chemosensitizing effect of azacitidine and vorinostat combination in seven patients enrolled in our study. Although five of these patients had disease refractory to several prior lines of therapy and all seven progressed on the study regimen, two patients had a complete response to subsequent treatment while three others had a significant clinical benefit. Further cellular and mice studies suggested that this effect could be consequence of the DNMTI rather than to the combination, since the addition of vorinostat to decitabine did not increase significantly the chemosensitizing effect of decitabine alone. Caution should be exercised when interpreting these results. The number of patients included in this experience is small, and the patients were heterogeneous with respect to their response to treatments administered before and after the clinical trial. Patients that appeared to derive the most benefit from azacitidine priming could have been those with less aggressive or more chemosensitive disease. Similarly, given the differences in duration of exposure to the study drugs and intensity of post-treatment therapy, it is not possible to extrapolate our experience to optimize the sequencing of DNMTI and chemotherapeutic agent. Also, a significant potential for increased toxicity exists, particularly when DNMTI is administered with potent alkylating agents, and we do not recommend using this approach outside the context of a clinical trial. Nonetheless, our results suggest that epigenetic priming with DNMTI could be a potential therapeutic approach for RR-DLBCL patients in order to overcome chemotherapy resistance and improve their outcomes. Thus, further clinical trials are needed to confirm the value of such strategy.

## Conclusions

In sum, we demonstrated that combination of HDI and DNMTI has synergistic activity in vitro and exhibits higher antitumor activity than single agents in vivo in DLBCL pre-clinical models. However, the combination of azacitidine and vorinostat in RR-DLBCL patients showed limited clinical activity. Post-trial follow-up of some of these patients and in vivo murine models suggested a sensitizing effect of the combination to subsequent therapies. In further pre-clinical studies, we demonstrated that DNMTI is likely the main contributor to this effect in DLBCL.

## Additional files

Additional file 1: Table S1. Toxicity. Highest grade of treatment-emergent adverse events encountered at the four different dose levels. (DLs). DL1: azacitidine (AZA) 55 mg/m2 days 1–5 and vorinostat (VST) 300 mg BID days 1–7. DL2: AZA 75 mg/m2 days 1–5 and VST 200 mg BID days 1–7. DL3: AZA 55 mg/m2 days 1–5 and VST 300 mg BID days 1–14. DL4: AZA 75 mg/m2 days 1–5 and VST 200 mg BID days 1–14. (DOC 57.0 kb) Additional file 2: Table S2. Treatment response at the four different dose levels (DLs). DL1: Azacitidine (AZA) 55 mg/m2 days 1–5 and vorinostat (VST) 300 mg BID days 1–7. DL2: AZA 75 mg/m2 days 1–5 and VST 200 mg BID days 1–7. DL3: AZA 55 mg/m2 days 1–5 and VST 300 mg BID days 1–14. DL4: AZA 75 mg/m2 days 1–5 and VST 200 mg BID days 1–14. (DOC 39 kb)
