# Supplementary material for: Combinatorial epigenetic therapy in diffuse large B cell lymphoma pre-clinical models and patients
Source: Clin Epigenetics. 2016 Jul 22;8:79. doi: 10.1186/s13148-016-0245-y (PMC4957280; doi:10.1186/s13148-016-0245-y)
Supplement: Additional file 1: Table S1. — Toxicity. Highest grade of treatment-emergent adverse events encountered at the four different dose levels. (DLs). DL1: azacitidine (AZA) 55 mg/m2 days 1–5 and vorinostat (VST) 300 mg BID days 1–7. DL2: AZA 75 mg/m2 days 1–5 and VST 200 mg BID days 1–7. DL3: AZA 55 mg/m2 days 1–5 and VST 300 mg BID days 1–14. DL4: AZA 75 mg/m2 days 1–5 and VST 200 mg BID days 1–14. (DOC 57.0 kb) [file 13148_2016_245_MOESM1_ESM.doc]

| **Supplementary Table 1**: Toxicity. | | | | | | | | |
| --- | --- | --- | --- | --- | --- | --- | --- | --- |
|  | DL 1 (n=8) | | DL 2 (n=5) | | DL 3 (n=4) | | DL 4 (n=1) | |
|  | Grade I/II | Grade III/IV | Grade I/II | Grade III/IV | Grade I/II | Grade III/IV | Grade I/II | Grade III/IV |
| **Hematological** |  |  |  |  |  |  |  |  |
| Anemia | 6 | 1 | 4 | 1 | 4 | 0 | 0 | 1 |
| Thrombocytopenia | 3 | 1 | 2 | 3 | 1 | 3 | 0 | 1 |
| Leukopenia | 1 | 1 | 1 | 1 | 2 | 2 | 1 | 0 |
| Neutropenia | 2 | 1 | 1 | 0 | 1 | 1 | 0 | 0 |
| **Non-hematological** |  |  |  |  |  |  |  |  |
| Diarrhea | 3 | 0 | 1 | 1 | 1 | 0 | 1 | 0 |
| Nausea | 6 | 0 | 3 | 0 | 3 | 0 | 0 | 0 |
| Vomiting | 3 | 0 | 3 | 0 | 0 | 0 | 0 | 0 |
| Fatigue | 1 | 0 | 2 | 0 | 1 | 0 | 0 | 0 |
| Fever | 2 | 0 | 0 | 0 | 0 | 0 | 1 | 0 |
| Hypoglycemia | 2 | 0 | 2 | 0 | 2 | 0 | 1 | 0 |
| Hyperglycemia | 1 | 0 | 3 | 0 | 1 | 0 | 0 | 0 |
| Renal impairment | 2 | 0 | 4 | 0 | 1 | 0 | 0 | 0 |
| Hyperbilirubinemia | 1 | 0 | 1 | 0 | 0 | 0 | 1 | 0 |
| Raised ALP | 2 | 0 | 1 | 0 | 2 | 0 | 0 | 1 |
| Raised AST | 0 | 0 | 0 | 0 | 0 | 0 | 1 | 0 |
| Raised ALT | 0 | 0 | 0 | 0 | 0 | 0 | 1 | 0 |
| Thromboembolic | 0 | 1 | 0 | 0 | 0 | 0 | 0 | 0 |
| *Abbreviations*: ALP, alkaline phosphatase; AST, aspartate aminotransferase; ALT, alanine transaminase; DL, dose level. | | | | | | | | |
