# Supplementary material for: Combinatorial epigenetic therapy in diffuse large B cell lymphoma pre-clinical models and patients
Source: Clin Epigenetics. 2016 Jul 22;8:79. doi: 10.1186/s13148-016-0245-y (PMC4957280; doi:10.1186/s13148-016-0245-y)
Supplement: Additional file 2: Table S2. — Treatment response at the four different dose levels (DLs). DL1: Azacitidine (AZA) 55 mg/m2 days 1–5 and vorinostat (VST) 300 mg BID days 1–7. DL2: AZA 75 mg/m2 days 1–5 and VST 200 mg BID days 1–7. DL3: AZA 55 mg/m2 days 1–5 and VST 300 mg BID days 1–14. DL4: AZA 75 mg/m2 days 1–5 and VST 200 mg BID days 1–14. (DOC 39 kb) [file 13148_2016_245_MOESM2_ESM.doc]

| **Supplementary Table 2**: Treatment response at the four different dose levels (DLs). | | | | | | | |
| --- | --- | --- | --- | --- | --- | --- | --- |
| Dose Level  (DL) | Progressive  Disease  (PD) | Stable  Disease  (SD) | Partial  Response  (PR) | Complete  Response  Unconfirmed  (CRu) | Complete  Response  (CR) | Not  Evaluated  (NE) | Total |
| DL1 | 5 | 2 | - | - | - | 1 | 8 |
| DL2 | 3 | 1 | 1 | - | - | - | 5 |
| DL3 | 3 | - | - | - | - | 1 | 4 |
| DL4 | 1 | - | - | - | - | - | 1 |
